# Tumor necrosis factor alpha, citrullination, and peptidylarginine deiminase 4 in lung and joint inflammation

**DOI:** 10.1186/s13075-016-1068-0

**Published:** 2016-07-22

**Authors:** Mandar Bawadekar, Annette Gendron-Fitzpatrick, Ryan Rebernick, Daeun Shim, Thomas F. Warner, Anthony P. Nicholas, Lennart K. A. Lundblad, Paul R. Thompson, Miriam A. Shelef

**Affiliations:** Department of Medicine, University of Wisconsin-Madison, Madison, WI USA; Research Animal Resource Center Comparative Pathology Lab and Department of Pathobiological Sciences, School of Veterinary Medicine, University of Wisconsin-Madison, Madison, WI USA; Department of Pathology and Laboratory Medicine, University of Wisconsin-Madison, Madison, WI USA; Department of Neurology and Center for Neurodegeneration and Experimental Therapeutics, University of Alabama at Birmingham and Birmingham VA Medical Center, Birmingham, AL USA; Department of Medicine, The University of Vermont, Burlington, VT USA; Departments of Biochemistry and Molecular Pharmacology, University of Massachusetts Medical School, Worcester, MA USA; Department of Medicine, University of Wisconsin-Madison and William S. Middleton Memorial Veterans Hospital, Madison, WI USA

**Keywords:** Rheumatoid arthritis, Citrullination, Peptidylarginine deiminase 4, Lung, TNF-α, Experimental arthritis

## Abstract

**Background:**

The relationship between lung and joint inflammation in rheumatoid arthritis is poorly understood. Lung inflammation with resultant protein citrullination may trigger anti-citrullinated protein antibodies, inflammation, and arthritis. Alternatively, lung and joint inflammation may be two manifestations of a single underlying pathology. The lung has increased citrullination and TNF-α levels are high in rheumatoid arthritis; however, it is unknown if TNF-α can induce lung protein citrullination. The citrullinating enzyme peptidylarginine deiminase 4 (PAD4) exacerbates TNF-α-induced arthritis, but a role for PAD4 in lung citrullination and TNF-α-induced lung inflammation has not been explored. Our aim was to use TNF-α-overexpressing mice to clarify the intersection of TNF-α, citrullination, PAD4, arthritis, and lung inflammation.

**Methods:**

Lung protein citrullination in wild-type mice, mice that overexpress TNF-α systemically (TNF^+^), TNF^+^PAD4^+/+^, and TNF^+^PAD4^-/-^ mice was quantified by both gel electrophoresis using a citrulline probe and western blot. Hematoxylin and eosin (H&E)-stained lung sections from TNF^+^PAD4^+/+^ and TNF^+^PAD4^-/-^ mice were scored for lung inflammation. H&E-stained ankle joint sections from mice that overexpress TNF-α only in the lungs were assessed for arthritis.

**Results:**

TNF^+^ mice have increased lung protein citrullination. TNF^+^PAD4^-/-^ mice do not have significantly reduced lung protein citrullination, but do have decreased lung inflammation compared to TNF^+^PAD4^+/+^ mice. Mice that overexpress TNF-α only in the lungs do not develop arthritis.

**Conclusions:**

PAD4 exacerbates lung inflammation downstream of TNF-α without having a major role in generalized protein citrullination in inflamed lungs. Also, TNF-α-induced lung inflammation is not sufficient to drive murine arthritis.

**Electronic supplementary material:**

The online version of this article (doi:10.1186/s13075-016-1068-0) contains supplementary material, which is available to authorized users.

## Background

Over the past decade, our understanding of rheumatoid arthritis has grown with a potential role for citrullinated proteins and the peptidylarginine deiminases (PADs) in disease development. Rheumatoid arthritis may start with an environmental trigger leading to protein citrullination. Genetically susceptible individuals form anti-citrullinated protein antibodies (ACPAs) as part of a loss of tolerance to citrullinated and other posttranslationally modified proteins. As inflammation increases secondary to the loss of tolerance, the levels of inflammatory cytokines such as tumor necrosis factor alpha (TNF-α) rise and ultimately inflammatory arthritis develops.

Citrullination is a posttranslational modification catalyzed by the PAD enzymes whereby arginines are converted to citrullines. PAD4 is particularly interesting in rheumatoid arthritis for several reasons. It is expressed in immune cells and is increased in the rheumatoid joint [1, 2]. Also, PAD4 can citrullinate proteins as part of immune-mediated membranolytic pathways [3] and as part of neutrophil extracellular trap (NET) formation [4–6], both of which have been implicated in ACPA development. Furthermore, single nucleotide polymorphisms in the gene encoding PAD4 are associated with rheumatoid arthritis [7, 8]. Therefore, PAD4 is likely important in the pathogenesis of human rheumatoid arthritis. Mice deficient in PAD4 have reduced TNF-α-induced [9] and glucose-6-phosphate isomerase-induced arthritis [10], but it is not known if PAD4 impacts lung citrullination or inflammation.

Lung inflammation is tightly linked to rheumatoid arthritis with clinical interstitial lung disease occurring in about 10 % of rheumatoid arthritis patients and subclinical lung disease in up to 80 % of rheumatoid arthritis patients [11]. Also, people with serum ACPAs and/or rheumatoid factor and no arthritis have twice the rate of subclinical lung disease as people without these autoantibodies [12]. Finally, citrullinated proteins are found in lungs and joints that are inflamed for a variety of reasons [13–16]. Based on these observations, two hypotheses emerge for how lung and joint inflammation are linked. One possibility is that lung and joint inflammation are two independent outcomes of the same pathologic loss of tolerance. In this model, citrullination could be induced in both tissues by the same factors, possibly by TNF-α given its ability to stimulate the formation of NETs [4]. Alternatively, lung inflammation with resultant protein citrullination may trigger ACPA development and ultimately rheumatoid arthritis. This latter hypothesis is consistent with observations that several risk factors for rheumatoid arthritis and ACPAs are inhaled [17, 18]. Additionally, in ACPA-positive rheumatoid arthritis patients, ACPA levels are higher in bronchiolar lavage fluid compared to serum, although synovial fluid ACPA levels were not assessed [13]. One limitation to most human studies focused on lung and joint inflammation is that they are correlative. Thus, an understanding of how lung and joint inflammation intersect has been elusive.

To test causality, it is often necessary to use animal models. One good model of rheumatoid arthritis is the TNF-α transgenic mouse, in which human TNF-α is overexpressed systemically causing a severe inflammatory and destructive arthritis that starts at 4–8 weeks of age depending on the number of copies of the TNF-α transgene [19, 20]. These mice also develop lung inflammation since overexpression of TNF-α in the lung induces a severe lung disease with alveolitis, fibrosis, emphysematous changes, endothelial changes, and platelet trapping [21, 22]. Given the presence of arthritis as well as both airway disease and interstitial lung disease (both of which have links to rheumatoid arthritis [23, 24]) induced by TNF-α, this model is particularly useful for exploring the pathophysiology of rheumatoid arthritis and rheumatoid lung disease. TNF-α-overexpressing mice have increased citrullination of serum proteins as well as autoantibodies that bind native and citrullinated proteins [9], but protein citrullination in the inflamed lungs of these mice has not been assessed.

In this study, we employ mice that overexpress TNF-α to determine if TNF-α can induce lung citrullination, if PAD4 is required for lung citrullination and/or lung inflammation downstream of TNF-α, and if TNF-α-induced lung inflammation is sufficient to induce arthritis.

## Methods

### Animals

Mice that overexpress systemically one copy of the TNF-α transgene (Tg3647 transgenic mice referred to here as TNF^+^ [19]) were crossed with PAD4-deficient mice [5] to generate TNF^+^PAD4^+/+^ and TNF^+^PAD4^-/-^ mice. Littermates were used for all experiments. Mice that overexpress TNF-α only in the lungs under the control of the surfactant promoter [21] and wild-type littermates were allowed to age 85–94 weeks.

### Protein lysates

Lung tissue was harvested from mice, flash frozen and homogenized using a Bullet Blender (Next Advance, Averill Park, NY, USA) in RIPA buffer supplemented with protease inhibitor cocktail (Sigma-Aldrich, St. Louis, MO, USA). Lysates were sonicated briefly and total protein was quantified.

### Detection of citrullination with rhodamine-phenylglyoxal (Rh-PG)

Equal amounts of protein were diluted with trichloroacetic acid and incubated with Rh-PG as previously described [25]. The reaction was quenched with L-citrulline, washed with acetone, and resuspended in 2× SDS loading dye for gel electrophoresis. Gels were imaged (excitation 532 nm, emission 580 nm) using an ImageQuant LAS 4000 (GE Healthcare Life Sciences, Chicago, IL, USA), stained with brilliant blue G – colloidal solution (Sigma-Aldrich, St. Louis, MO, USA) and re-imaged using a LI-COR Odyssey infrared scanner (LI-COR Biosciences, Lincoln, NE, USA). Full lane densitometry for Rh-PG and brilliant blue was performed.

### Detection of citrullination by western blot

Equal amounts of protein were subjected to gel electrophoresis using two polyacrylamide gels. One gel was stained with brilliant blue and the other was transferred to a polyvinylidene difluoride membrane (Bio-Rad, Hercules, CA, USA) using a semi-dry blotting system (Bio-Rad, Hercules, CA, USA). The membrane was blocked with milk, incubated overnight at 4 °C in 1:1000 mouse monoclonal anti-peptidyl citrulline (F95) antibody [26] in milk, washed, incubated with a 1:10,000 dilution of anti-mouse IgM conjugated to IRDye 680LT (LI-COR Biosciences, Lincoln, NE, USA) for 1 hour at room temperature, washed, and imaged using the Odyssey infrared scanner followed by whole lane densitometry. Findings are unchanged when densitometry is performed excluding bands that are the same size as those seen using anti-mouse IgM alone.

### Pathology

Lungs and hind legs were fixed in 10 % neutral-buffered formalin, processed with a VIP processor, embedded in paraffin (Tissue Tek, Sakura, Torrance, CA, USA), sectioned and stained with hematoxylin and eosin (H&E). Hind legs were decalcified for 30 hours with Surgipath Decalcifier 1 (Leica Biosystems, Wetzlar, Germany) prior to processing and embedding. Lung disease was scored in a blinded manner by a board-certified pathologist on a scale of 0–5 (0, none; 1, mild; 2, mild to moderate; 3, moderate; 4, moderate to severe; 5, severe) for the following characteristics: lymphocytic parabronchiolar foci, perivascular lymphoid infiltrates, destructive vasculitis, interstitial inflammatory infiltrates, and alveolar infiltrates. Scores were not limited to integer values. Ankle joints were evaluated for synovitis and erosions in a blinded manner by a board-certified pathologist.

### Statistics

All data were analyzed using a *t* test with the results considered significant if the two-tailed *p* value was less than 0.05.

## Results

### TNF-α induces lung citrullination

**Fig. 1:**
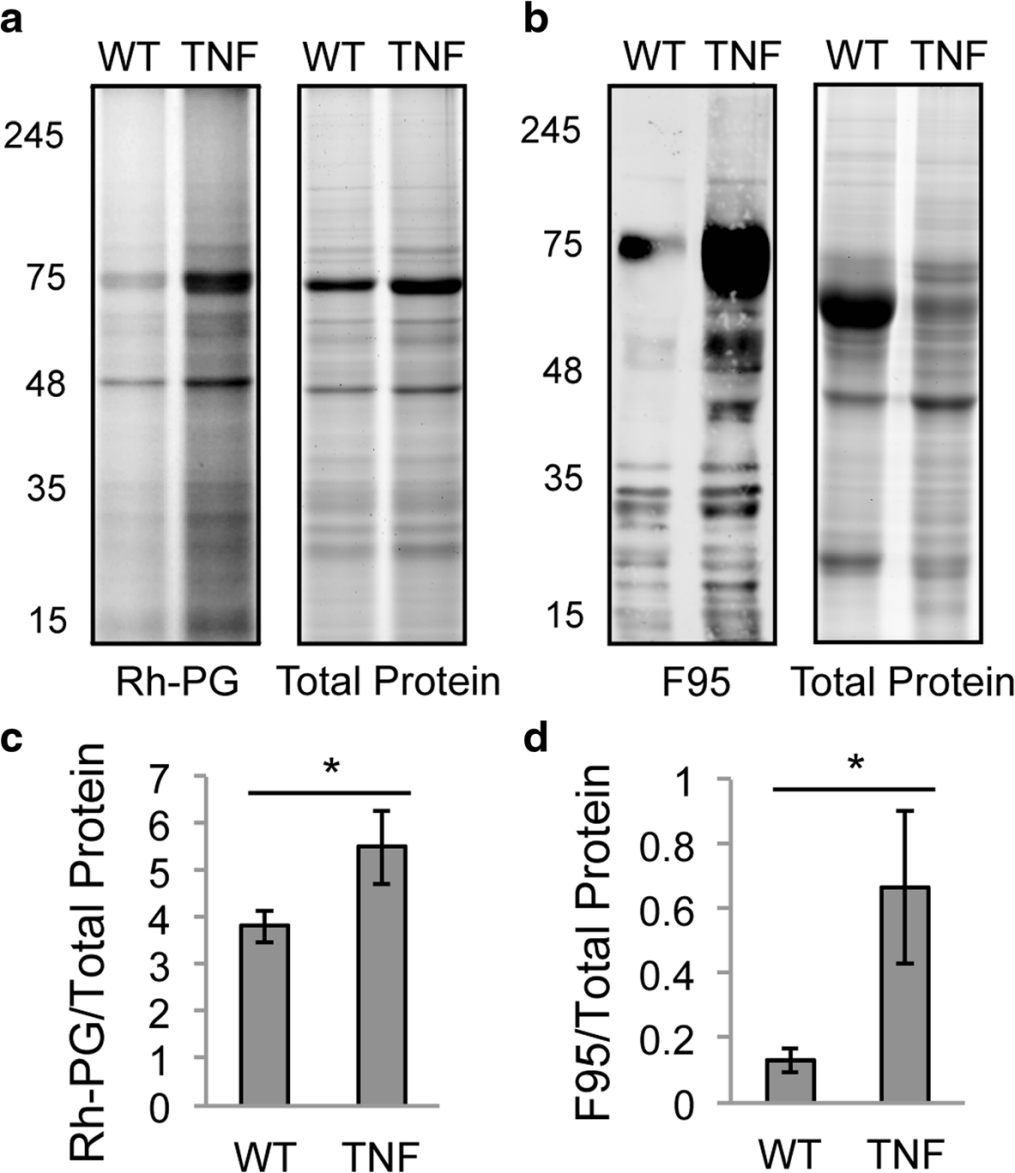
TNF-α induces lung citrullination. Lung protein lysates from TNF-α overexpressing (TNF) and control (WT) mice were exposed to Rh-PG followed by gel electrophoresis, imaging of Rh-PG, then staining with brilliant blue to detect total protein. **a** Representative gels. **b** Total Rh-PG signal was normalized to total protein with average and SEM graphed. Lysates were subjected to western blot using the F95 antibody to detect citrullinated proteins and gel electrophoresis with brilliant blue to detect total protein. **c** Representative western blot (*left*) and brilliant blue-stained gel (*right*). **d** Total F95 signal was normalized to total protein with average and SEM graphed. For all panels, n = 5 TNF and 6 WT, ^*^ *p* < 0.05 *TNF-α* tumor necrosis factor alpha, *WT* wild-type

To quantify protein citrullination, we allowed a citrulline-specific, fluorescently labeled chemical probe, Rh-PG [25], to bind to protein followed by gel electrophoresis to visualize the Rh-PG-bound protein. After confirming increased Rh-PG binding to in vitro citrullinated fibronectin [27] compared to native fibronectin (see Additional file 1), we used Rh-PG to assess citrullination in lung lysates from 5-month-old mice that overexpress TNF-α systemically (TNF^+^ mice) and wild-type littermates. As shown in Fig. 1a and b, there is increased total protein citrullination in lung lysates from TNF^+^ compared to wild-type mice. No significant increase in protein citrullination is seen in TNF^+^ compared to wild-type mice at 2 and 3.5 months of age (data not shown).

Although normal lungs have some baseline PAD activity [28] and native fibronectin may have some baseline citrullination, we were concerned about potential background signal using Rh-PG. Further, Rh-PG detects carbamylated proteins reducing its specificity. Thus, we repeated our experiments using a monoclonal anti-peptidyl-citrulline antibody (F95) to detect citrullinated protein by western blot [26]. First, we confirmed increased binding of F95 to citrullinated fibronectin as compared to native fibronectin by western blot (see Additional file 1). Then, we used F95 to assess protein citrullination in the lungs of TNF^+^ and control mice by western blot. As shown in Fig. 1c and d, TNF^+^ mice have increased lung protein citrullination compared to wild-type littermates at 5 months of age. No significant increase in citrullination was seen at 2 and 3.5 months of age in TNF^+^ mice using F95 (data not shown). Taken together, our data suggest that TNF-α induces citrullination in murine lungs.

### PAD4 is not required for lung citrullination in TNF^+^ mice

**Fig. 2:**
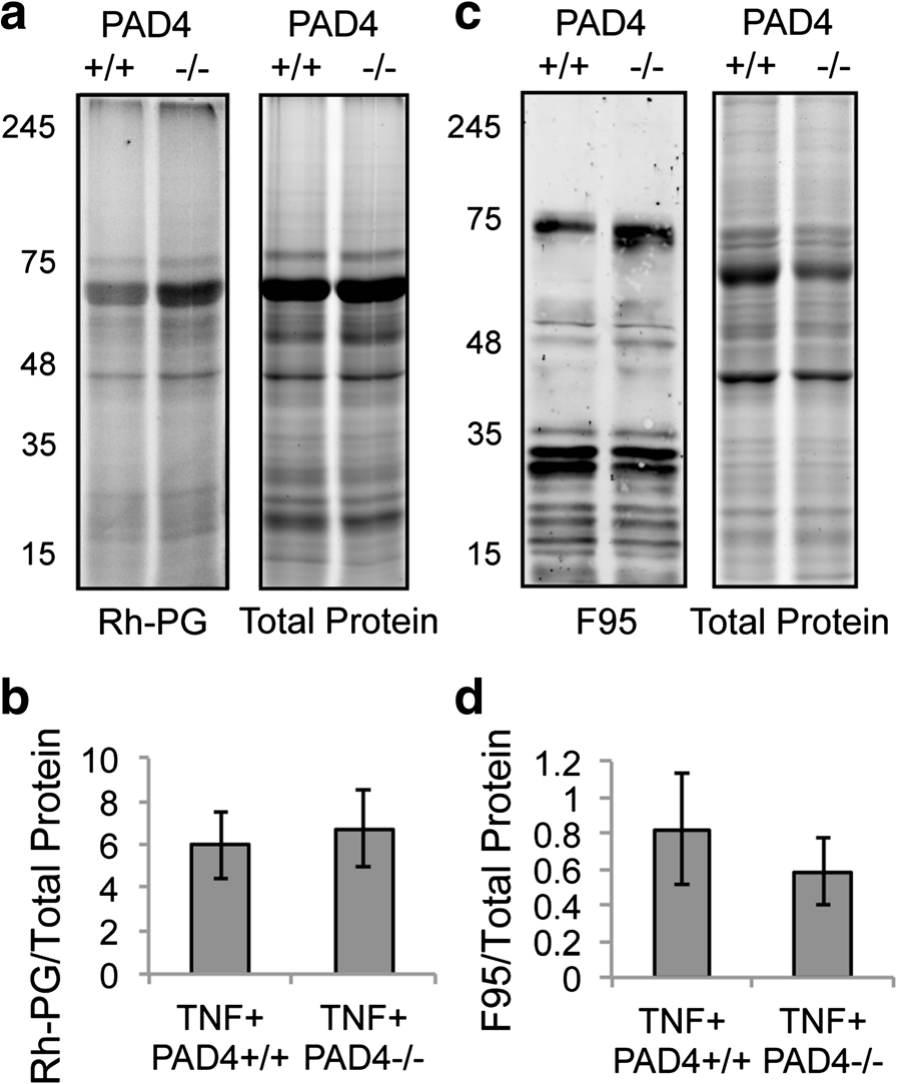
PAD4 is not required for TNF-α-driven lung citrullination. Lung lysates from TNF^+^PAD4^+/+^ and TNF^+^PAD4^-/-^ mice were exposed to Rh-PG, followed by gel electrophoresis, imaging of Rh-PG, then staining with brilliant blue to detect total protein. **a** Representative gels. **b** Total Rh-PG signal was normalized to total protein with average and SEM graphed. Lysates were subjected to western blot using the F95 antibody to detect citrullinated proteins and gel electrophoresis with brilliant blue to detect total protein. **c** Representative western blot (*left*) and brilliant blue-stained gel (*right*). **d** Total F95 signal was normalized to total protein with average and SEM graphed. For all panels, n = 9 littermate pairs. *PAD4* peptidylarginine deiminase 4, *TNF-α* tumor necrosis factor alpha

After demonstrating that lung citrullination is increased in TNF^+^ mice, we wanted to determine if the citrullination seen might require PAD4. Therefore, we used Rh-PG as above to quantify protein citrullination in lung protein lysates from 5-month-old TNF^+^PAD4^+/+^ and TNF^+^PAD4^-/-^ mice. We did not detect a reduction in total protein citrullination in the lung (Fig. 2a and b) in TNF^+^PAD4^-/-^ mice compared to TNF^+^PAD4^+/+^ mice. To support these results, we performed western blots on lung lysates as above using F95. In agreement with the Rh-PG results, we saw no significant reduction in total protein citrullination in the lung in TNF^+^PAD4^-/-^ mice compared to TNF^+^PAD4^+/+^ mice (Fig. 2c and d). Similar results were seen in the lungs of TNF^+^PAD4^-/-^ and TNF^+^PAD4^+/+^ mice at 2 and 3.5 months of age using both methods (data not shown). Also, we did not detect a significant reduction in citrullination of specific protein bands in TNF^+^PAD4^-/-^ compared to TNF^+^PAD4^+/+^ mice (data not shown). These data suggest that PAD4 is not critical for gross protein citrullination in TNF^+^ mice.

### PAD4 contributes to TNF-α-induced lung inflammation

**Fig. 3:**
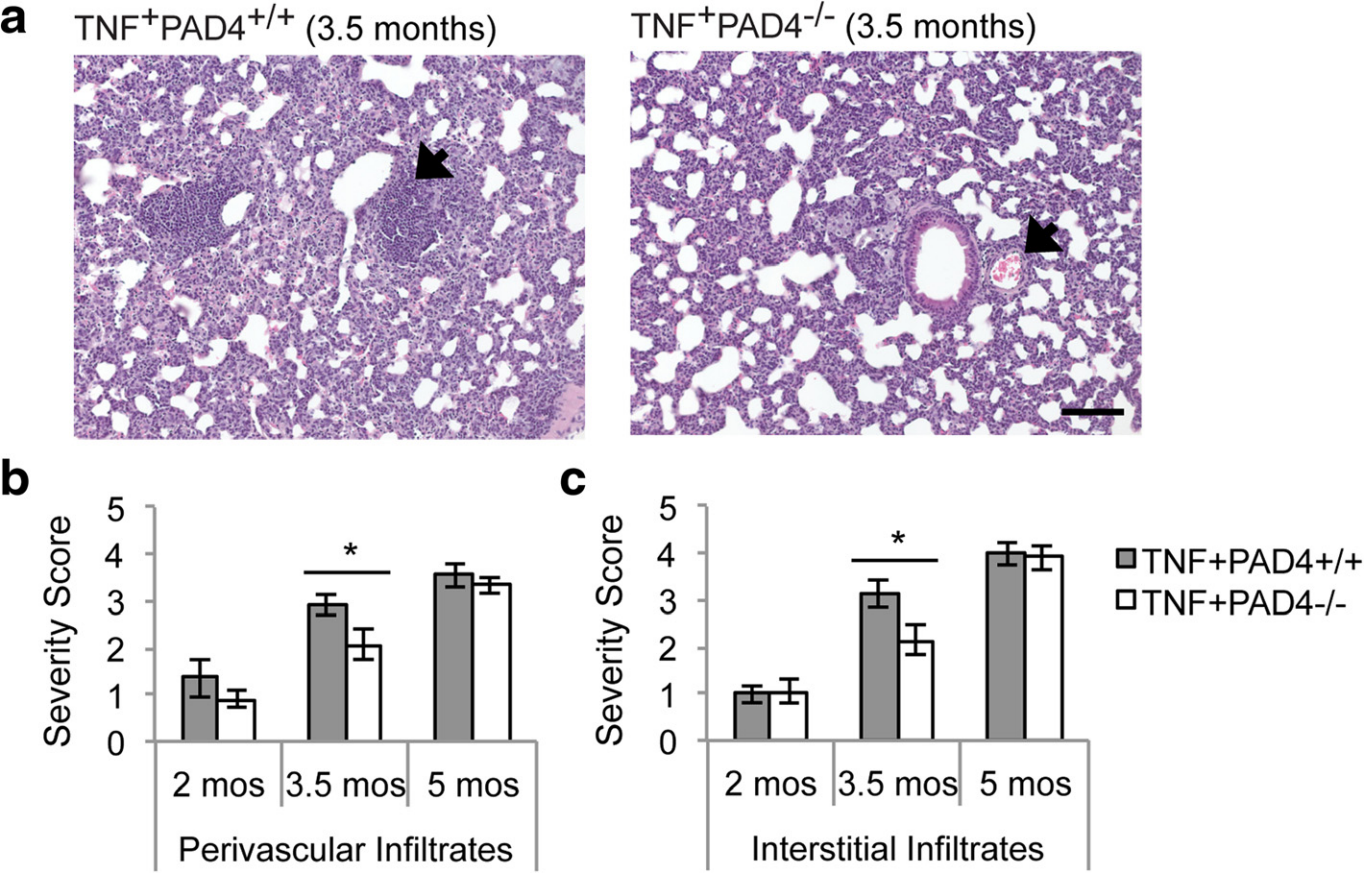
PAD4 contributes to inflammatory infiltrates in TNF-α-driven lung inflammation. H&E-stained lung sections from 2-, 3.5-, and 5-month-old TNF^+^PAD4^+/+^ and TNF^+^PAD4^-/-^ littermates were scored for the severity of perivascular infiltrates and interstitial infiltrates. **a** Representative images of TNF^+^PAD4^+/+^ and TNF^+^PAD4^-/-^ lungs at 3.5 months of age (200×, bar 0.1 mm). *Arrows* indicate representative perivascular lymphoid infiltrates. Average and SEM for severity of perivascular infiltrates (**b**) and interstitial infiltrates (**c**) are shown (2 months old: n = 5 littermate pairs, 3.5 months old: n = 6 TNF^+^PAD4^+/+^ and 7 TNF^+^PAD4^-/-^ mice, 5 months old: n = 10 TNF^+^PAD4^+/+^ and 11 TNF^+^PAD4^-/-^ mice; ^*^ *p* < 0.05). *PAD4* peptidylarginine deiminase 4, *TNF-α* tumor necrosis factor alpha

**Fig. 4:**
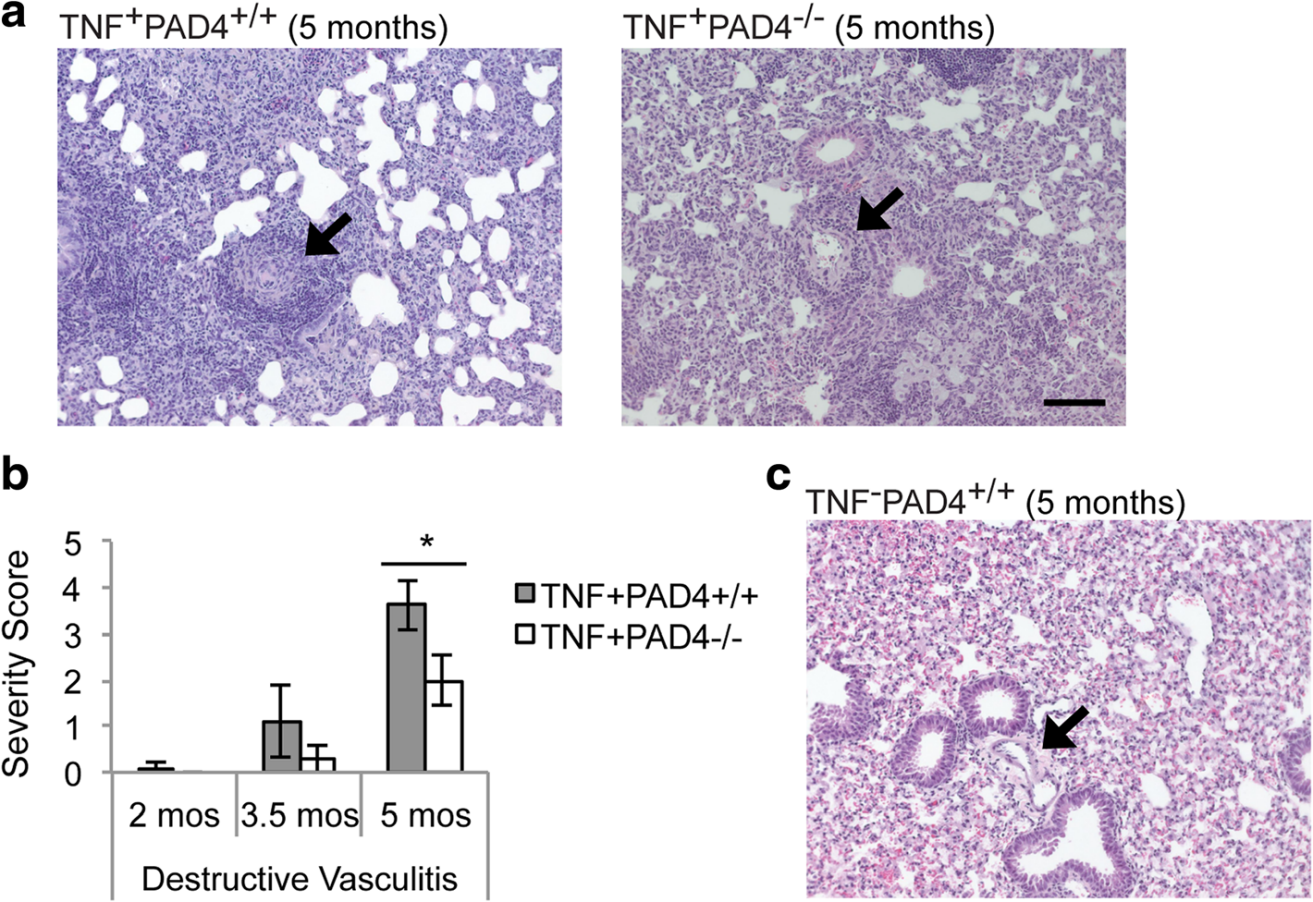
PAD4 contributes to vasculitis in TNF-α-driven lung inflammation. H&E-stained lung sections from 2-, 3.5-, and 5-month-old TNF^+^PAD4^+/+^ and TNF^+^PAD4^-/-^ littermates were scored for the severity of destructive vasculitis. **a** Representative images of TNF^+^PAD4^+/+^ and TNF^+^PAD4^-/-^ lungs at 5 months of age. *Arrows* point to vessels with representative amounts of destructive vasculitis. **b** Average and SEM for severity of destructive vasculitis are shown (2 months old: n = 5 littermate pairs, 3.5 months old: n = 6 TNF^+^PAD4^+/+^ and 7 TNF^+^PAD4^-/-^ mice, 5 months old: n = 10 TNF^+^PAD4^+/+^ and 11 TNF^+^PAD4^-/-^ mice, ^*^ *p* < 0.05). **c** Five-month-old wild-type littermate lung. *Arrow* points to representative normal vessel. All images: 200×, bar 0.1 mm. *PAD4* peptidylarginine deiminase 4, *TNF-α* tumor necrosis factor alpha

Despite our observation that PAD4 is not critical for total lung citrullination in TNF^+^ mice, we hypothesized that PAD4 might be important for TNF-α-induced lung inflammation since it contributes to TNF-α-induced joint inflammation [9]. To test this hypothesis, H&E sections from the lungs of 2-, 3.5-, and 5-month-old TNF^+^PAD4^+/+^ and TNF^+^PAD4^-/-^ mice were scored for the severity of lung disease in a blinded manner. At 3.5 months of age, TNF^+^PAD4^-/-^ mice have reduced perivascular lymphoid infiltrates and interstitial infiltrates compared to TNF^+^PAD4^+/+^ mice (Fig. 3). The severity of both perivascular and interstitial infiltrates increases at 5 months of age and the difference between TNF^+^PAD4^+/+^ and TNF^+^PAD4^-/-^ mice disappears. However, by 5 months of age, a destructive vasculitis becomes prominent and this vasculitis is reduced in TNF^+^PAD4^-/-^ compared to TNF^+^PAD4^+/+^ mice (Fig. 4a and b). Five-month-old controls that do not overexpress TNF-α (TNF^-^PAD4^+/+^) show no inflammatory infiltrates or destructive vasculitis (Fig. 4c). There was no difference between TNF^+^PAD4^+/+^ and TNF^+^PAD4^-/-^ mice with regards to parabronchiolar inflammation or alveolar infiltrates at any age (data not shown). Taken together, these data suggest that PAD4 contributes to interstitial and perivascular inflammation as well as destructive vasculitis downstream of TNF-α in murine lungs.

### TNF-α overexpression in the lung is not sufficient to induce arthritis in mice

**Fig. 5:**
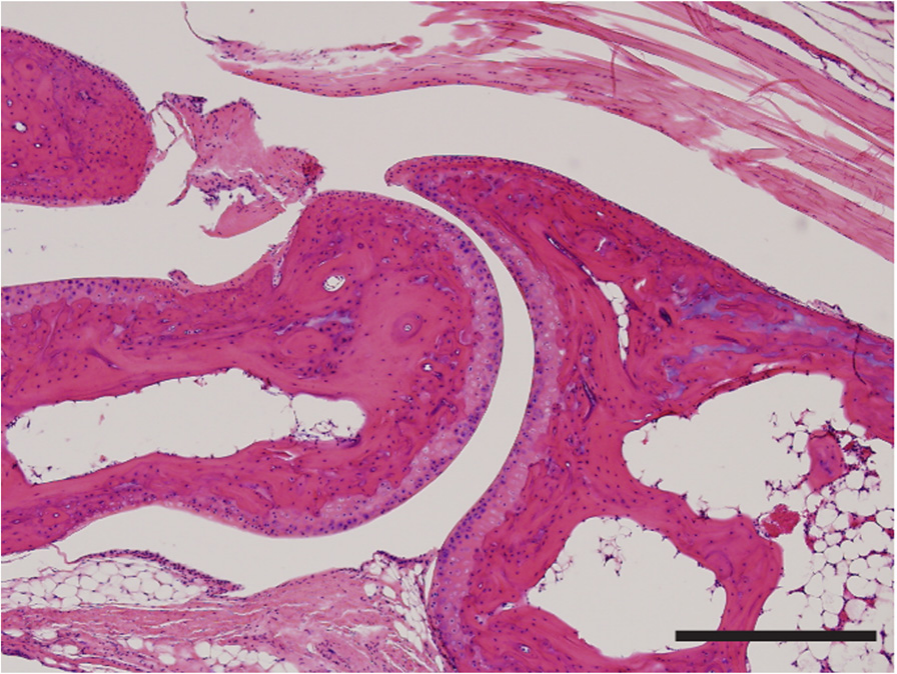
TNF-α-driven lung inflammation is not sufficient to induce arthritis. Ankle joint sections from 21- to 22-month-old mice that overexpress TNF-α only in the lungs under the control of the surfactant promoter and wild-type controls were H&E stained. Representative image is shown (*n* = 3 lung TNF and 4 WT, 100×, bar 0.2 mm). TNF-α tumor necrosis factor alpha, WT wild-type

Since TNF-α induces protein citrullination in the lung, which is thought to be a potential trigger for rheumatoid arthritis, we hypothesized that TNF-α-induced lung inflammation might induce arthritis development. Therefore, we harvested the joints from almost 2-year-old mice that overexpress TNF-α only in the lungs under the control of the surfactant promoter [21]. We prepared H&E sections and then evaluated for the presence of arthritis in a blinded manner. None of the mice that overexpress TNF-α only in the lung had any arthritis by histology similar to wild-type controls (Fig. 5). Thus TNF-α-driven lung disease is not sufficient to induce inflammatory arthritis in mice.

## Discussion

In the present study, we show that overexpression of TNF-α, a major pathologic cytokine in rheumatoid arthritis, induces murine lung citrullination providing evidence for a pathway by which lung citrullination may be induced in human disease. In human rheumatoid arthritis, high levels of TNF-α may be a trigger for the observed citrullination in the rheumatoid lung [16]. The presence of citrullinated proteins in the lung downstream of TNF-α may provide fodder for inflammation, particularly in ACPA-positive individuals, thus contributing to ongoing disease.

Despite our observation that TNF-α induces lung citrullination, we found that TNF-α-driven lung inflammation is not sufficient to induce arthritis. Our work builds on previous studies, which found that chronic exposure to tobacco smoke or intratracheal bleomycin is not sufficient to drive arthritis in SKG mice [29], a murine line that develops lung and joint inflammation upon exposure to microbial products [30, 31]. Taken together, these studies do not support a model in which lung inflammation independently induces joint inflammation. Rather, joint inflammation and lung inflammation appear to be two manifestations of a single pathologic process. In the case of our experiments, the pathologic process is high levels of TNF-α, which is also seen in rheumatoid arthritis.

The same mechanism may be true in humans. Once tolerance is lost, increasing levels of inflammatory cytokines like TNF-α in the preclinical phase of rheumatoid arthritis may drive both joint and lung inflammation. It is known that cytokines rise prior to the onset of arthritis [32], but less is known about the chronology of cytokine levels and lung inflammation. However, one cannot simply translate murine findings to human rheumatoid arthritis for several reasons. First, there are genetic differences between humans and mice, particularly in the major histocompatibility complex (MHC) class II locus. Specific MHC class II variants are closely associated with ACPAs and rheumatoid arthritis [33] and bind citrullinated peptides better than native peptides [34]. The absence of specific MHC variants may explain the absence of autoantibodies that uniquely target citrullinated antigens in mice that overexpress TNF-α [9] as well as possibly the inability for TNF-α-induced lung inflammation to independently drive murine arthritis. Also, humans may have exposure to decades of lung inflammation or “second hits” such as smoking, minor joint trauma, or infections that could drive arthritis, none of which our mice experience. Thus, it is possible that the lung could play an inciting role and also be a target organ in human rheumatoid arthritis. However, our data only support the role of the lung as a target and not as an initiator of arthritis.

We have also found that PAD4 does not play a major role in lung citrullination in TNF^+^ mice. This result is surprising given previous studies that show a relationship between TNF-α, nuclear localization of PAD4, citrullination, and rheumatoid arthritis [14, 35–37]. However, we used two different methods to assess protein citrullination, which gave similar results. Also, our findings are in agreement with previous work showing that PAD4 is not required for citrullination of serum proteins in TNF^+^ mice as detected by a colorimetric assay [9]. Thus, although PAD4 is capable of citrullinating proteins, it does not appear to be required for a large portion of lung protein citrullination downstream of TNF-α. It is possible that PAD4 is required for the citrullination of a small number of proteins or specific arginines in a protein [38], but gross protein citrullination in the lungs of TNF^+^ mice, which is similarly seen in the rheumatoid lung [16], is not critically dependent on PAD4. Thus, it is likely that other PAD enzymes, such as PAD2, are responsible for total lung citrullination downstream of TNF-α. Like PAD4, PAD2 is expressed in immune cells [2, 38]. Further, PAD2 is increased in the lungs of smokers [39, 40] and the synovial fluid of ACPA-positive rheumatoid arthritis patients [41]. PAD2 is also required for baseline PAD activity in non-inflamed lungs [28]. Further studies are needed to determine if PAD2 is required for TNF-α-induced citrullination.

We have also shown that PAD4 is important for TNF-α-driven lung inflammation in agreement with previous findings that PAD4 contributes to TNF-α-driven joint inflammation [9]. Initially PAD4 contributes to perivascular and interstitial inflammatory infiltrates and later to destructive vasculitis in the lung. Of note, patients with rheumatoid arthritis can develop a systemic vasculitis. Our findings suggest that PAD4 could contribute to rheumatoid vasculitis, which would be consistent with observations that PAD4 and NETs may be involved in other forms of vasculitis [42, 43].

Our finding that PAD4 significantly contributes to lung inflammation, but not total protein citrullination in the lung, supports a role for PAD4 apart from mass antigen citrullination. This role is likely multifactorial since, in addition to a role for PAD4 in neutrophils, PAD4 is expressed in monocytes and macrophages [2] and it contributes to the presence of activated T cells (including Th17 cells) and total immunoglobulin levels in murine inflammatory arthritis [9, 10]. Further, treatment with PAD inhibitors reduces inflammatory cytokine production by HL60 cells [44] and induces apoptosis in lymphoid cells [45]. The observed reduction in perivascular lymphoid infiltrates in TNF^+^PAD4^-/-^ lungs points to a role for PAD4 in the lymphoid compartment, although we cannot conclude if PAD4 is required in lymphocytes or in cells that regulate lymphocytes. PAD4 may have large effects on immune cells while citrullinating only a few total arginines. Such a small amount of citrullination would likely not be detected with our methods. Nonetheless, PAD4 appears to contribute to inflammation independently of gross citrullination. Additional studies are needed to better characterize the role of PAD4 in immune cells, clarify the role of PAD2 in citrullination and arthritis, and determine how both PADs contribute to human rheumatoid arthritis.

## Conclusions

We have shown that PAD4 contributes to lung inflammation, but is not critical for gross protein citrullination in the inflamed lung. These findings suggest that PAD4 exacerbates inflammation apart from antigen citrullination and that PADs other than PAD4 are responsible for the majority of inflammation-dependent protein citrullination in the lung. Further, we have shown that TNF-α-induced lung inflammation is not sufficient to cause murine arthritis, suggesting that lung and joint inflammation are two separate outcomes of a single underlying pathologic process instead of lung inflammation driving murine inflammatory arthritis. Further work is needed to extrapolate these findings to human rheumatoid arthritis.

## Abbreviations

ACPA, anti-citrullinated protein antibody; MHC, major histocompatibility complex; NET, neutrophil extracellular trap; PAD, peptidylarginine deiminase; TNF-α, tumor necrosis factor alpha

## Additional file

Additional file 1: Figure S1. Increased Rh-PG and F95 binding to citrullinated fibronectin. Fibronectin purified from human blood was citrullinated in vitro as previously described [27]. Native (FN) and citrullinated (cit-FN) fibronectin were exposed to Rh-PG followed by gel electrophoresis, imaging of Rh-PG, and staining with brilliant blue to detect total protein. **a** Representative gels. **b** Average and SEM are graphed for total Rh-PG signal normalized to total protein. FN and cit-FN were subjected to western blot using the F95 antibody and gel electrophoresis with brilliant blue to detect total protein. **c** Representative blot (*upper*) and gel (*lower*). **d** Average and SEM are graphed for total F95 signal normalized to total protein. For all panels, n = 3 experiments, ^**^ *p* < 0.01, ^***^ *p* < 0.001. (PDF 1349 kb)
