# Supplementary material for: Tumor necrosis factor alpha, citrullination, and peptidylarginine deiminase 4 in lung and joint inflammation
Source: Arthritis Res Ther. 2016 Jul 22;18:173. doi: 10.1186/s13075-016-1068-0 (PMC4957385; doi:10.1186/s13075-016-1068-0)
Supplement: Additional file 1: Figure S1. — Increased Rh-PG and F95 binding to citrullinated fibronectin. Fibronectin purified from human blood was citrullinated in vitro as previously described [27]. Native (FN) and citrullinated (cit-FN) fibronectin were exposed to Rh-PG followed by gel electrophoresis, imaging of Rh-PG, and staining with brilliant blue to detect total protein. a Representative gels. b Average and SEM are graphed for total Rh-PG signal normalized to total protein. FN and cit-FN were subjected to western blot using the F95 antibody and gel electrophoresis with brilliant blue to detect total protein. c Representative blot (upper) and gel (lower). d Average and SEM are graphed for total F95 signal normalized to total protein. For all panels, n = 3 experiments, ** p < 0.01, *** p < 0.001. (PDF 1349 kb) [file 13075_2016_1068_MOESM1_ESM.pdf]

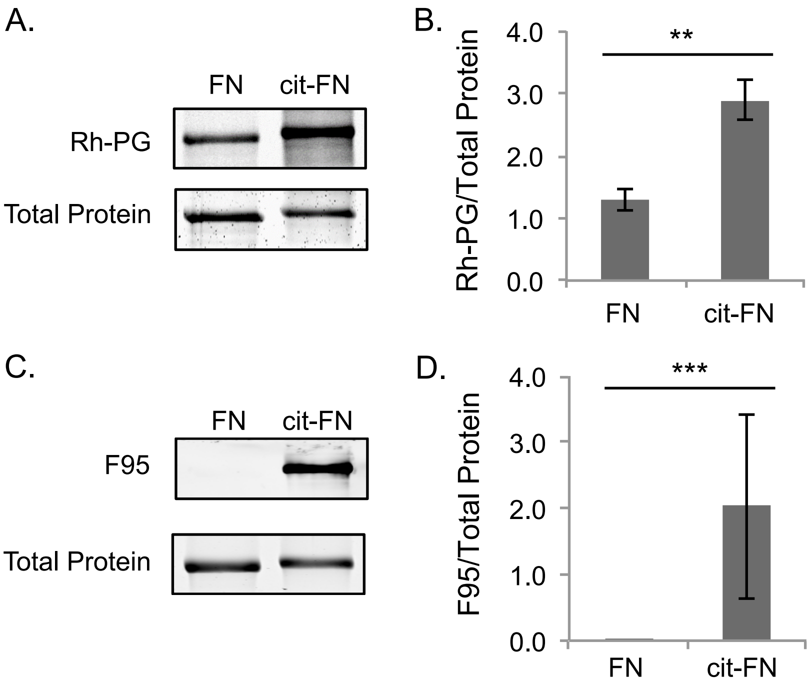

Supplemental Figure 1. Increased Rh-PG and F95 binding to citrullinated fibronectin. Fibronectin purified from human blood was citrullinated *in vitro* as previously described [26]. Native (FN) and citrullinated (cit-FN) fibronectin were exposed to Rh-PG followed by gel electrophoresis, imaging of Rh-PG, and staining with brilliant blue to detect total protein. A. Representative gels. B. Average and SEM are graphed for total Rh-PG signal normalized to total protein. FN and cit-FN were subjected to western blot using the F95 antibody and gel electrophoresis with brilliant blue to detect total protein. C. Representative blot (upper) and gel (lower). D. Average and SEM are graphed for total F95 signal normalized to total protein. For all panels, n=3 experiments, **p<0.01, ***p<0.001.
